# Supplementary material for: Cell-Penetrating Peptide Modified PEG-PLA Micelles for Efficient PTX Delivery
Source: Int J Mol Sci. 2020 Mar 9;21(5):1856. doi: 10.3390/ijms21051856 (PMC7084196; doi:10.3390/ijms21051856)
Supplement: Supplementary file 1 [file ijms-21-01856-s001.pdf]

Cell-penetrating peptide modified PEG-PLA micelles for efficient PTX delivery

Qi Shuai [1,ǂ], Yue Cai [1,ǂ], Guangkuo Zhao[1] and Xuanrong Sun [1,*]

[1] Collaborative Innovation Center of Yangtze River Delta Region Green Pharmaceuticals, Zhejiang University of Technology, Hangzhou 310006, China; qshuai@zjut.edu.cn (Q.S.); caiyue1992@foxmail.com (Y.C); (Z.G.);
* Correspondence: sunxr@zjut.edu.cn; Tel.: +86-57188871566
ǂ Equal contributors

1. Synthesis of Maleimide-PEG2000-PLA

1.1. Synthesis of HOOC-PEG2000-OH.

7.5 mmol PEG (15.0 g) was dissolved in 150 mL dichloromethane (DCM). Then 15 mmol anhydrous pyridine (1.2 mL, 2 eq), 0.75 mmol DMAP (0.092 g, 0.1 eq), and 7.5 mmol succinic anhydride (0.75 g, 1.5 eq) were added into the solution and reacted with PEG under the protection of nitrogen. After 12 h of reaction, another 3.75 mmol of succinic anhydride (0.375 g, 0.5 eq) was added into the reaction solution and stirred for another 12 h. The mixture was then washed twice with 0.1 M HCl and saturated brine. The organic phase was dried over Na_2SO_4, evaporated and finally added dropwise into 250 mL 4 °C diethyl ether to purify the products. White powders were collected by filtration and dried under vacuum.

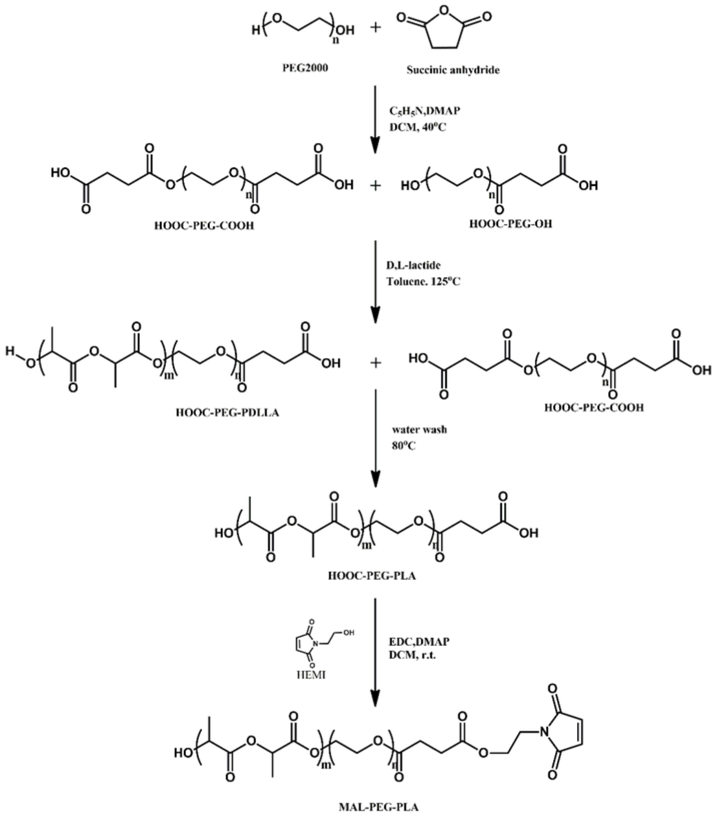

Figure S1. Synthesis of mal-PEG-PLA

1.2. Preparation of HOOC-PEG-PLA.

2 g of the mixture (HOOC-PEG-OH and HOOC-PEG-COOH), 11.1 mmol D, L-lactide (1.6 g) were added into 35 mL toluene and the solution was dried by distilling off 20 mL toluene. Then, stannous octoate (0.05 wt %) was used as catalyst to conduct the polymerization for 5 h at 125 °C under the protection of nitrogen. After cooled, the product was then diluted with 10 mL DCM, instilled in 200 mL 4 ◦C ether and gathered by filtration. The finally purified copolymers were dried in a vacuum for 24 h and then dissolved in 20 mL 80 °C deionized water with stirring until a thick oil phase precipitated at the bottom.[1] Then the supernatant which contained HOOC-PEG-COOH was removed by a glass burette, and another 10 mL 80 °C deionized water was added again with stirring. The whole procedure was repeated three times to completely remove water-soluble HOOC-PEG-COOH. Finally, 1.3 g of white powder was gained as a target block polymer after lyophilization.

1.3. Preparation of mal-PEG-PLA.

0.24 mmol of obtained polymer (1 g), 0.96 mmol HEMI (137 mg, 4 eq), 0.96 mmol EDC·HCL (184 mg, 4 eq) and 0.021 mmol DMAP (2.9 mg, 0.1 eq) were dissolved in 5 mL DCM, and stirred under nitrogen atmosphere at room temperature. After 72 h of reaction, the resulting mixture was washed by 0.1 M HCl (10 mL × 2) and saturated brine (10 mL × 2). The DCM phase was dried over Na_2SO_4, concentrated and added dropwise into 100 mL 4 ◦C ether, followed by filtration. Finally, 0.89 g of polymer was obtained and further dried under vacuum.

2. Preparation of Nanoparticles

15 mg TAT peptide and 2.5 mg TCEP were dissolved in 5 mL of 0.01 M HEPES (pH =7.0) buffer and stirred for 0.5 h. Then 330 mg of mal-PEG-PLA was added and reacted with TAT peptide for another 8 h. The final copolymer of TAT-PEG-PLA was washed twice by ultrafiltration (Mw = 3500) and finally collected after lyophilization.

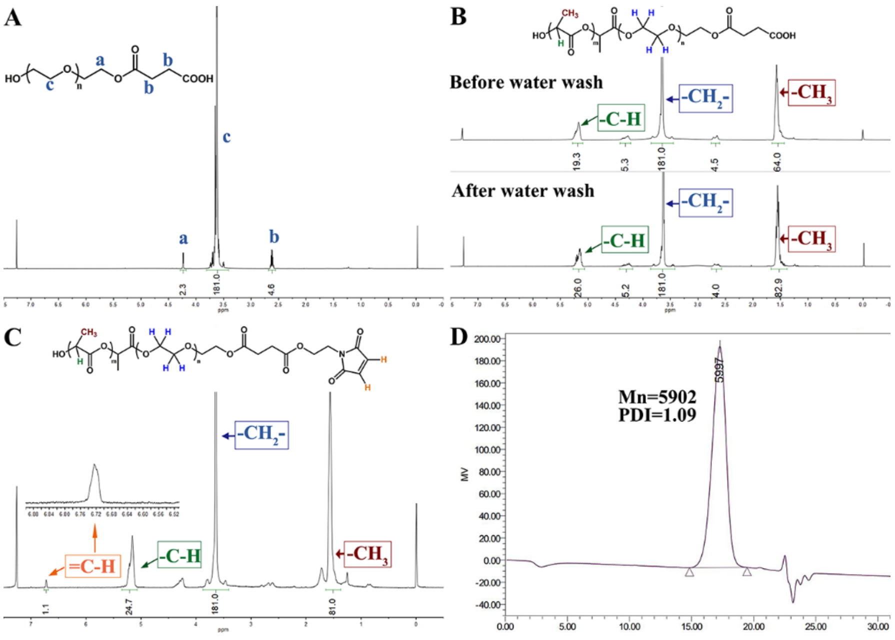

Figure S2. Characterization of all synthesized polymers. (A) 1H NMR spectra of the mixture of HOOC-PEG-COOH and HOOC-PEG-OH; (B) 1H NMR spectrum of the mixture of HOOC-PEG-PLA and HOOC-PEG-COOH before and after water wash. The molecular weight of the PLA block in the HOOC-PEG-PLA increased form 1460 Da to 1930 Da after washing with hot water, indicating that the HOOC-PEG-COOH

has been removed; (C) 1H NMR spectrum of mal-PEG-PLA; (D) Characteristic GPC trace of the purified mal-PEG-PLA.

TAT conjugated PTX-loaded nanoparticles (TAT-NP-PTX) were prepared by dialysis method. Briefly, 2 mg PTX and 50 mg TAT-PEG-PLA were dissolved in 1 mL DMSO, and the organic solution was slowly pipetted into 10 mL deionized water with stirring. After stirring at room temperature for 2 h, the solution was loaded into a dialysis bag (Mw = 3500) to completely remove DMSO via dialysis against pure water (4 L × 3) for 24 h. After dialysis, the remained free PTX was removed by a 220 nm filter. Meanwhile, the drug-loaded PEG-PLA nanoparticles (NP-PTX) were also prepared as the same method described above using mal-PEG-PLA.

Coumarin-6 loaded nanoparticles (NP-C6) and TAT conjugated Coumarin-6-loaded nanoparticles (TAT-NP-C6) were prepared in the same way just replaced PTX with C6. Briefly, 50 mg TAT-PEG-PLA or PEG-PLA was dissolved in 1 mL DMSO which containing 0.1 mg Coumarin-6. Then the mixture was slowly added dropwise into 10 mL deionized water with stirring. After stirring at room temperature for 2 h, the solution was loaded into a dialysis bag (Mw = 3500) to completely remove DMSO via dialysis against pure water (4 L × 3) for 24 h. After dialysis, the remained free Coumarin-6 was removed by a 220 nm filter and the micelles were obtained by lyophilization.

Reference

1. Yang, J.; Yan, J.; Zhou, Z. H.; Amsden, B. G., Dithiol-PEG-PDLLA Micelles: Preparation and Evaluation as Potential Topical Ocular Delivery Vehicle. *Biomacromolecules* **2014,** 15, (4), 1346-1354. https://doi.org/10.1021/bm4018879
